# Miscellanies

**Published:** 1839-04-01

**Authors:** 


### MISCELLANIES.

### A New Weekly Contemporary.

Start not gentle editors of the antipodes of Medical Journalism—ye of the Gazette and Lancet. We do not propose to turn hebdomadal, nor do we announce a rival. Your interests are safe at present. Still you will be spared the verification of the adage, that, "though two are company, three are none." Dr. Ryan is not resuscitated, nor has Henderson of the Old Bailey, obtained a respite for some tenant of the condemned cell on condition that he should work for life in irons. Some modest gentleman has not yet discovered that all your matter is mere moonshine, and your correspondents so many huffers—that practical knowledge is most found where there is least practice—that acquaintance with physic is not to be sought among the regular "saw-bones," but in those fortunate physicians, who, having been bred to something else, turn doctors when the other trade has failed—that an enormous capital is requisite for criticism, and proves it by displaying a stock of impertinence that would suffice for all the daily papers—in short, that a new journal is absolutely requisite, and that he is *the* man who has just the proper quantum and no less, of judgment and wit, of learning and fun, of strict impartiality and undeviating infallibility. No Katerfelto of this description has yet sent the bellman round, and our weekly contemporaries of London may still continue to exist.

The new Journal is a Paddy. He comes direct from Dublin, and "His legs that would make a chairman stare," display an Irish breadth and length, refreshing to see after the shankless Cockneys.

The "Dublin Medical Press," is our hero's name, given, we have no doubt, on the Hibernian principle, by which Bill was called Robinson—"for shortness." Suppose, readers, we tell Patrick to bow and address your honours. He is well up in the blarney :—

"It is impossible, by mere assertions, to prove the sincerity of professions, or to remove the suspicion that men act from interested motives only—but let the readers put themselves in the place of the editors, and then see whether their position is so enviable, or the prospect before them so flattering as might at first sight be supposed. Great labour, pecuniary risk, and personal obloquy are sure to be encountered; the return for which, it must be admitted, is not necessarily the most gratifying. In seeking the support of the profession, therefore, they are almost induced rather to claim it as a right than to supplicate it as a favour. Every man who can think must admit that something of this kind is required, and that the want of it has been a positive injury to every individual. In the country, gentlemen have been kept in total ignorance of what is going on in the metropolis, or are misled by imperfect information or misrepresentation. In Dublin, they are equally ignorant of the real state of the profession in the provinces. Year after year information of the utmost value has been lost, from the want of an efficient vehicle for its effectual diffusion, to which all, without repugnance or hesitation, could have recourse. Emulation has ceased, and a deplorable apathy has been engendered by the absence of competition and the want of the stimulus of example. The rights of individuals and the interests of the profession at large have been compromised or sacrificed from the want of a medium through which their complaints could be conveyed, or their wants made known, in an authenticated form, to the legislature or the public. The managers of many public institutions, uninfluenced by public opinion, refuse to keep pace with the advance of improvement. The business of instruction and the regulation of education is interrupted, because those interested in the continuance of irregularities have found that they could proceed without danger of detection or reproval."

It is evident how much the Dublin Medical Press is wanted. That being the case, its success is certain, or political economy is a flam. The demand regulates the supply, and the Dublin Medical Press shews us that the demand is pressing. The only draw-back on the glowing representation, is the remarkable circumstance, that, owing we suppose to the perversity of human nature, no sooner does a public-spirited editor furnish a Journal, which, on his own unexceptionable shewing, exactly takes the measure of the breechless public, than another editor starts another Journal, with the same lamentation on the same want, and the same declaration that he can remedy it. Nay it is a curious fact that the more Journals there are the more necessity would there seem to be for fresh. Were there none there would be merely a vacuity to fill. But those existing are so bad, that the rubbish must first be cleared away, before the process of hole-stopping can even be entered on. We have remarked, and it is a striking fact, that the more popular a Journal is, and the larger sale it has, the more indignant is the remonstrance of the new comer, and the more glaring does he represent the want of a periodical to be—a striking instance of the blindness of the public and discrimination of editors.

We may presume then that a new weekly Journal is much desired in Dublin. Let us hear what our young friend, the Medical Press, has to say for his intentions. Is he high Tory, Conservative, Whig, Whig-radical, Tory-radical, or out and outer? What has he to say on our *Corn*-laws—what on the *Charter*? We find that there has been deep solicitude on the subject in Ireland.

"In order to relieve the anxiety of our friends, and the apprehensions of our enemies, it is necessary to say a few words as to the tone and temper we hope to be able to adopt and preserve in the course of our labours. Satisfied that a free press, especially when directed to educated and cultivated minds, is the most powerful means in existence to elicit truth, encourage honesty, bridle folly, and resist oppression, we propose to avail ourselves of its powers to the fullest extent to which it is legitimately applicable. So far from concurring in the opinion entertained by many, that the free public discussion of medical affairs is to be dreaded, we are firmly convinced that it is the only means by which justice can be obtained, confidence restored, and those dissensions, heartburnings, and collisions, which distract and divide the profession, removed. Nevertheless, entertaining these views, we are equally convinced that confining this power within its proper limits is a work of the utmost difficulty; and that while we shall hereafter study to attain this object, we shall, we fear, necessarily occasionally fail of success.

From all observation bordering on interference with the private or professional affairs of our brethren, it is, we hope, unnecessary to say we feel bound scrupulously to refrain; but, on the other hand, we must remind our readers that when a man challenges remark, by voluntarily presenting himself or his opinions to public attention, he must take the consequences in good humour. Certain spoiled children of fortune, habituated to the deference of a small circle of dependents, and flattered by themselves and their admirers into a very unreasonable estimate of their sagacity and disinterestedness, view with impatience and anger all expression of doubt as to their sincerity, or hesitation as to their capacity or judgment. When such men come forward to influence public measures, attempting to persuade others as they have persuaded themselves, that they are impelled by the loftiest sentiments of regard for the public good, they are mortified and irritated if any suspicion be entertained that the ruling passion weakens their judgment or warps their opinions. This expression of doubt in their sincerity they call imputing motives. Such doubts and suspicions we must, as journalists, entertain; and if the expression of them be called imputation of motives, we must stand convicted of that offence. In undertaking the duty we have imposed on ourselves we assume that we are the retained advocates of the rights of the profession, and the interests of the medical institutions in particular, and that we are bound to resist and expose every attempt, direct or indirect, to encroach on the one or sacrifice the other."

There are one or two points on which we feel doubt. A free press and free discussion, says our Editor, will remove dissensions. If they do, we will go to school to our contemporary, and take lessons in editing. Neither a free press nor a shackled one has ever lulled party-spirit yet, nay, the general hypothesis is, that the freedom of the press is conducive to faction. It has its advantages, but making people unanimous is not one of them.

Another subject of misgiving is, our friend being retained both for the profession and the institutions. How he means to serve God and Mammon is not clear. But, on this side of the Channel, the Irish colleges are a complete piece of botheration to us. We know nothing about them, and the wheels within wheels are neither seen nor understood. The Irish pures, for instance, are wroth at not being allowed to sell medicines. But we come to more tangible matters. It seems, after all, that our quarterly contemporary has said and done, that even his "sad and learned" writers have not found out the secret of reviewing. Yet that is odd. The public must recollect our contemporary's prospectus. Sir Benjamin Brodie was to do all the articles upon the joints, at the rate of a guinea a line—Sir Astley Cooper was hired for the thymus gland—Dr. Baron was retained expressly for cow-pox—and Goss and Co. were engaged for the seminal weakness department. The editors had stationed themselves in different quarters of the country, merely to watch the progress of science, (how could they do that if they were fixtures in town ?) and make meteorological observations. Yet in spite of all this, authors were not satisfied—all kinds of reclamations were made—partiality, prejudice, and ignorance were openly charged on editors who had proclaimed the reign of abstract purity and of universal knowledge—and, in short, reviewing is as bad as ever. The Dublin Press, however, will set matters right.

"With respect to the manner of conducting our reviewing department it is necessary to say, that we hope to be able to avoid the two extremes which constitute the great reproach of the periodical criticism of the day. While we shall study to avoid the fulsome language of overstrained panegyric, which disgusts by its exaggeration, we shall also study to avoid all wanton outrage of the feelings of writers by a display of our critical attainments at their expense. Statements or reasonings calculated to lead to erroneous conclusions must, of course, be denied or refuted ; but we hold that in doing so it is unnecessary to indulge in harsh expressions or arrogantly assume a superiority to which, after all, we may not be entitled. With style or language we have nothing to do. If bad, we cannot mend it ; if good, the reader will discover its beauties without our assistance. An author often loses sight of his subject while he endeavours to polish a sentence or round a period ; while he who writes at once what he knows and thinks, succeeds, because he is intent only on securing a belief in his statements. Those anxious for the diffusion of knowledge should not discourage the exertions of a labourer in the field, because his efforts are feeble or his method awkward. The pecuniary loss sustained by publishing of a bad book, and the loss of character incurred by the author, are sufficient punishment, while the work itself 'mixes with the stream and strengthens the general current.' There is, however, a class with which we shall not hesitate to deal freely : those who write, not because they have anything of value to communicate, or because they are anxious to contribute to the improvement of the rising generation, but because they want notoriety, and seek to keep their names before the public eye. They are to us like the hawkers of damaged wares through the streets, we are sickened and stunned by their vociferations. Those also who by accident or management have been placed in the position of public instructors, and who, reckless of all consequences, use the privilege conferred on them without judgment or discretion, must be arrested in their career. The practice of dictating without adequate authority, drawing hasty conclusions from insufficient data, and attaching undue value to mere novelties, has done incalculable mischief to the student. There is another class, also, with which we must occasionally come into collision; but our persuasions shall be more in sorrow than in anger. These are the sentimental admirers of the modest pretensions of the Parisian school, who pertinaciously propagate the delusion that from this source only is knowledge to be derived. We hope to compel them to prove or abandon their position. Our reviews, from our limited space, must be brief, more analytical notices than elaborate essays, informing our readers that certain works have been published, and acquainting them with their contents and probable value."

The "Press" is determined to have some fun. Why should curing folks be melancholy work? Why should reviewers be doggedly dull. Here again the Dublin hits our contemporary. The solemn dulness of his leaden page is too heavy for the mercurial temperament of Ireland.

"In what humour shall we address our readers? Our sad and learned brethren will counsel seriousness and reprove all levity subversive of the acknowledged gravity of the medical functionary. We ourselves incline the other way. We see no reason why we should not sweeten labour by a little play, where the opportunity offers. We have enough of scenes to encounter calculated to engender melancholy—let us not create new causes of it. We must occasionally indulge mirth, even at the risk of shocking our sedate friends; and while we borrow a leaf from an Hippocrates or a Galen, sometimes enliven it by a sally from a Rabelais or an Arbuthnot. Such weapons cannot be considered unlawful, although to be used with caution and good feeling. If provoked to mirth by weakness, vanity, or overweening presumption, it shall not be displayed by a sardonic grin, or ironical sneer, but a correcting smile, or that privileged expression of amusement which extravagance justifies."

Our Irish contemporary is right. There is a time for all things, and assuredly there is not a more certain sign of the absence of genius, than a disrelish for humour and wit. But a truce to what Baxter calls the "heavy-assed Christians."*

Our Dublin friend will pardon our badinage. We shake hands all the more cordially, because we do so with a laugh. We wish our weekly contemporary success, and, until he can run alone, which we hope and believe will be very shortly, we shall feel the greatest pleasure in giving him a lift. All we insist on is—a sincere desire to advance science, and the absence of those personal attacks which are happily passing away from the pages of medical journals. Publications, actions, humbug, affectation are fair game.

---

### Professional and Gentlemanly bearing of the American Medical Press.

The Medical Examiner, a journal at present published every fortnight, and soon to be published every week, in Philadelphia, makes the following observations on the tone of the medical press of America.

* "A shove to Heavy-assed Christians."

"The tone and spirit of the American medical press is, in general, unexceptionable. The freest expression of opinion is shown to be compatible with decorum and courtesy of language. It is felt that personalities are unworthy the dignity of our professional literature, and if, here and there, an outbreak of ill-temper and scurrility emanates from some turbulent spirit, the contempt awarded him proves the general correctness of taste and feeling.

Sectional feeling is, we are glad to say, seldom evinced by the professional periodicals of our country. The appeal to local prejudices, in a literary discussion, is paltry. It degrades the scientific to the level of the partisan political journal, and, though it may succeed in effecting particular private ends, it is visited with the disapprobation of every candid and enlightened mind. Happily, our remarks have no application to the majority of our contemporaries. If they have some point, in a solitary instance, for the honour of the profession, we regret it. We trust that the party in question will profit by the rebuke of a journal of its own vicinity; and, for the future, abstain from 'introducing this species of unjust and unmanly, or, to say the least, *unscientific* writing into a medical journal.'"

So far as our acquaintance with our Transatlantic contemporaries goes, the portrait is perfectly correct. They are distinguished by the absence of that truculent spirit which has infested and disgraced the medical press of this country. The editors of these republican journals evince a degree of courtesy which some affect to belong exclusively to aristocratic society. Of the Medical Examiner itself, we can speak in high terms. We are confident, from the manner in which it is conducted, that success will wait upon its labours.

---

### Temperance Societies.

These societies were first formed in North America, at Boston, in 1813. The abuse of spirituous liquors was first forbidden, but the use of them was permitted. The interdiction was eluded, and for twelve or thirteen years little was accomplished. In 1826 many influential inhabitants of Boston united to form one, in which the use of spirituous liquors should be totally interdicted. In 1828 there were 222 such societies, numbering 30,000 persons. In 1829 a diminution of the mortality was observed in persons under 40 years of age. In 1831 the use of spirits was put a stop to in the American army. The following year 500 vessels left the American ports without spirits on board, and the rates of insurance were lowered on this account. On board vessels of war, sailors relinquishing the allowance of grog received an equivalent. The troops received an equivalent in sugar, coffee, and rice. In 1834 the temperance union of the United States was formed at Philadelphia, to serve as a general centre for the other societies. Vessels adopting the temperance plan made more rapid and prosperous voyages than others—and in 1835, of 186 whaling vessels, 168 took no spirituous liquors; and the insurance was lowered five per cent. in consequence. This year two millions renounced the use of spirituous liquors, four thousand distilleries were discontinued, eight thousand persons discontinued the trade in spirits, more than twelve hundred captains sailed without spirits on board their vessels, and twelve thousand habitual drunkards were cured. The first temperance society in Europe was established in 1829, at New Ross, in Ireland; and others were established shortly after, as well in Ireland as in Scotland. In 1830 they were established in Sweden, Finland, and some parts of Russia. In 1831 the London temperance society was established, with the Bishop of London at its head; and many have been established in the colonies.—*Annales d'Hygiene Publique, Oct.* 1838. *Dublin Medical Press, No.* 1.

These are flattering results of the water *minus* rum system. Vessels on the temperance plan made more " rapid and prosperous" voyages, than the groggy ones. Nature, herself would appear to have joined the temperance society; cool and comfortable, she only puffed a steady breeze upon the swelling canvas of the tea-totallers, while the perverse lovers of grog were always in a calm or a hurricane.

Then twelve thousand *habitual drunkards* were cured. Not a soul less than this glorious number was redeemed. It was not eleven thousand nine hundred and ninety-nine—No, it was exactly twelve thousand. Think of that Master Brook. We never saw a habitual drunkard *cured*. Yet here are twelve thousand recoveries. Will any one laugh at tea-totalism after that?

The truth is, that this is a species of fanaticism, got up to meet the drunkard's fanaticism. It may do good, for many are ever in extremes, and can abstain when they cannot moderate. But the idea that tea-totalism will ever prevail extensively is quite chimerical. It may stop a gap, and keep a few of the million sober, until the diffusion of education and of knowledge, instructs the mass in their real interests.

---

### Sir Philip Crampton's History of Medicine.*

Our excellent, and witty friend has been reading an amusing and clever sketch of the History of Medicine before the Royal College of Surgeons of Ireland. It is very spirited, and by no means uninstructive. We shall extract a few passages from it. Sir Philip divides medicine historically thus:—

" 1stly. You have what may be called *natural or instinctive medicine*, in which every man was his own physician, and where there were no persons set apart as practitioners of the art; this is a state in which medicine exists to this hour in many regions of the earth, and even in this country there are many populous districts that are without any other medical aid, than that which the uneducated inhabitants supply to one another. In those remote districts, however, the clergy often perform the offices of the healing art, but in a very different spirit from that in which it was exercised by the priests who officiated in the temples of Esculapius.

2ndly. You had the ' medicina sacra,' or medicine as practised by the priests of whatever religion,

3rdly. You had ' philosophic medicine,' or medicine as it was practised in Greece, previously to the æra of Hippocrates, to whom Celsus ascribes the merit of having ' separated medicine from philosophy,' a merit to which I shall have occasion to shew he was not entitled.

Lastly, you have ' empirical medicine,' or medicine, very much as it is practised amongst us at this day, in which close attention is paid to the *signs* of disease, rather than to their *proximate causes*, and in which medicines are exhibited, with reference to their known *effects* rather than to their *occult qualities*. This custom of discarding hypotheses, as to the *causes* of disease, and the *modus operandi* of remedies, is not of very modern date, although it has been revived within almost our own times, and is confined almost to the British nation; it is exactly the doctrine of the empirical sect of which Serapion was the head, and which sprung up shortly after the death of Hippocrates."

2. *Division of Labour among the Egyptians.*—Those of our readers who have read Herodotus will probably recollect his stating that, in Egypt—every distinct distemper had its own physician, who confined himself to the study of that and of no other, so that all places were crowded with physicians; one class had the care of the eyes, and another of the head, another of the teeth, another of the stomach, and another of occult diseases. The Egyptians forestalled the

---

* Dub. Journ. Jan. 1839.

moderns. Your mad doctor, heart doctor, water doctor, and pox doctor are descendants of the ancient brood. In a high state of civilization these subdivisions of labour must take place.

3. *Pretensions to Anatomical Knowledge in the Time of Cicero.*—This extraordinary man appears to have considered the study of anatomy unnecesary. "The public," he says, "have a notion, that a knowledge of the internal structure of a man is useful to a physician, and hence it is, that the physicians *pretend* to possess it." But what little the physicians of those days did know of anatomy was obtained from animals and from *authority*. We dare say that many of our readers remember the saying of a comparatively modern anatomist, on finding that the human body did not correspond with plates of, we believe, Vesalius'—"the parts must have altered since his time."

4. *Some Cases of Cure by the Gods as well authenticated as any effected by Animal Magnetism.*—Sir Philip gives us this brief description of the mode of treatment adopted by the principal physicians of Greece, prior to the era of Hippocrates—the priests of Esculapius.

All who had the means of repairing to those temples were accommodated within its precincts: having in the first place laid their offerings on the altar, and been purified by the lustral water, they were put to rest in the middle of the temple, and as soon as they were supposed to be asleep, a priest clad in the vestments of Esculapius, and surrounded by a troop of damsels, who were devoted to the service of the temple, and were well instructed actresses, entered the sacred dormitory, for the purpose of indicating to the patient the remedy which the oracle of the deity had revealed as most suitable to his case. As the god could only reveal himself in a dream, the patients were placed on beds of ram's skins, which were supposed to cause divine visions, and it was considered an impiety for the patient to open his eyes, and not to feign a profound sleep, even though wide awake; and above all, to doubt that whatever he *did* hear with his ears, or see with his eyes, was other than a celestial vision.

The servant, into whose mouth Aristophanes puts this recital, describes, in a comical manner, the address and promptitude displayed by the chief priest in putting into his sack, while these ceremonies were being performed, all the votive offerings which had been laid upon the altar.

It seems to signify little what caste of men constitute the doctors. They all appear to have a strong disposition to *sack* the spoil. From Esculapius down to Dupotet, animal magnetism has had this at least substantial in its tendencies. The revelations at the North London Hospital are after all but a scurvy imitation of the Esculapian chorus, and had the latter been got up with proper accompaniments, we doubt whether it would not have *taken* better. Yet the Okeys, (we stand corrected the O'Keys) were clever pantomimists.

But the practice of the priestly mummers was far from unsuccessful. It was the custom of the patient, when cured, to hang up a votive tablet describing his case and the lucky treatment. Nothing could be more unexceptionable in the way of testimony. There it was, every one could see it, if false it could be contradicted, and not being contradicted of course it was true. An ancient tablet of this kind is preserved by the family of Maphæa. Sir P. Crampton copies from it one or two of the shortest cases—specimens of all.

1st case. The god pronounced the following oracle to Caius, who was blind, that he should approach the sacred altar, and having bent his knees, he should pass from the right to the left; after this, he should place his five fingers on the altar, then raising his hand he should apply it to his eyes, which, no sooner had he done, than he saw perfectly well; all the people present testified their joy that so great a miracle should have been performed in honour of our emperor Antoninus.

2nd. The god gave this oracle to a blind soldier, named Valerius Aper, that he should take the blood of a white cock, and mixing it with honey, use it as a collyrium; this he did for three days, and his sight was restored; he came to return thanks publicly to the god, and place this tablet in his temple.

A third is cured of a spitting of blood by eating apples, taken from the altar and stewed in honey.

We should like to know whether animal magnetism can produce any thing more satisfactory. Nothing but Esculapius or animal magnetism could effect such cures.

5. *The Claims of Hippocrates to admiration.*—"He had no suspicion that the brain was the organ of sensation and motion; he considered it, on the contrary, to be a gland or sort of great sponge, for the purpose of absorbing the pituita and the superfluous humours of the body, and discharging them through the nose. The heart he considered as the origin of the arteries and nerves (mistaking the cordæ tendinæ for nerves); and the liver as the origin of the veins. The lungs he considered as the absorbers and condensers of the humidity of the body. But this is, I trust, a sufficient sample of the state of anatomy and physiology in the time of Hippocrates; with respect to his practice it may be enough to say, that it was founded on hypothetical notions respecting the four humours (as he calls them) of the body—blood, pituita, yellow bile, and black bile. A state of health he considered to consist in the happy proportion in which these humours were combined; and disease, in the derangement of these proportions; then, much depended on the influence of the stars, and on the power of numbers. Seven days were allowed for the acute stage of fever, and seven for the chronic; during the first seven, little or no nourishment was to be given, but during the last, the coction of the humours being completed, the patient was to be amply supplied with food."

6. *There have always been plenty of St. John Longs and Ingestries.*—Every body knows how Lord Ingestrie deposed very solemnly that he saw St. John Long extract something very like mercury out of a man's head. It will be observed from the following statement of Rhases' that this kind of humbug is almost as ancient as animal magnetism itself.

"There are so many little arts used by mountebanks and pretenders to physic, that an entire treatise, had I a mind to write one, would not contain them. Some give out that they can draw snakes or lizards out of their patients' noses, which they seem to perform by putting up a pointed iron probe with which they wound the nostril till the blood comes, then they draw out the little artificial animal composed of the coagulated blood, or of liver, &c. Some pretend *that they can collect all the floating humours of the body to one place by rubbing it with* wild cherries, *which causes a burning or inflammation,* and they expect to be rewarded as if they had cured the distemper."

7. *The tragical End of the great Anatomist, Vesalius.*—This extraordinary man completed his astonishing work upon anatomy, in the year 1543, when he was yet only 27 years of age. When we consider, remarks our author, that when Vesalius commenced his labours, there was nothing extant on the subject of anatomy, but the wretched treatises of Galen, and the small work of Mundinus, giving the result of the dissections of three bodies, with what astonishment must we view a work so complete, that little of the anatomy of the human body, and that little absolutely unimportant, has been left unobserved. Unhappily Vesalius was in advance of his century. His story has been partly told in touching though in simple language by Ambrose Paré.

"Il ne faut point se hâter d'ensevelir et encore moins d'ouvrir le corps des femmes hysteriques de pour d'encourir une calomnie ainsi que de ce siècle est arrivé à un grand anatomiste; je dis grand et célèbre duquel les livres réparent aujord'hui les études des hommes doctes, lequel étant pour lors residant en Espagne fut mandé pour une femme de maison qu'on estimoit être morte par une suffocation de matrice. Le deuxième coup de rasoir qu'il lui donna commença ladite-femme à se mouvoir et à démontrir par autres signes quelle vivoit encore dont tous les assistans furent grandement étonés. Je laisse a penser au Lecteur, comme ce bon seigneur faisant cette œvres fut un peiplexété comme on cria *Tolle* apris lui, tellement que tout ce qu'il put faire fut de s'absenter du pays, car ceux qui le devoient excuser c'étoient ceux qui lui couroient sus, et étant exilé, tôt après mounet de déplaisir, qui n'a été sans une grande perte pour la republique."

The affair however, as Sir Philip tells us, had a different issue from that related by Paré. It appears, that pursued by the relations of the woman according to some, or of a nobleman (according to others,) whose trance he mistook for death, (although it is highly probable, that the story was got up for his destruction,) he was condemned to death by the tribunal of the Inquisition, but at the interposition of Philip II., whose physician he was, and by whom he was beloved, sentence of death was commuted to a pilgrimage to the Holy Land. Returning from Jerusalem, his vessel was wrecked on the Island of Zante, near the Morea, where he died of cold and hunger, on the 15th October, 1564.

8. *The Stone crushed in the Bladder* 270 *Years ago.*—The paper containing this fact is lodged in her Majesty's State Paper Office in Ireland, book iii. p. 259, February 1567. The patient was Sir Henry Sidney, Lord Deputy Lieutenant. "*The State of Sir H. Sidney's Bodie, MS., Ireland,* 1559, *in Her Majesty's Office of Papers and Records of State.*—My Lord President, being of the age of xxxvi. years, went into Ireland a hole man, not touched with the stone, and so remained one yeare and a half or thereabought, and then, after long grief, avoided two stones which were very big, such as few men have been known to have avoided. After this he took his journey into the north parts of Irelande, and so continued void of pain or grief until his arrival in Englande, which was about 8 weeks after, and then at Chester felt the like grief as at first, and so continued in pain until Christmas Eve; at that time being searched with surgeons he avoided one other stone, *broken by the surgeon his iustruments in divers pieces,* for that it was so great that otherwise it could not be taken out, for all the pieces laid together might make the quantity of a nutmegge."—*Collins's Lives and Actions of the Sidneys*, p. 95."

One cannot attach much value to so lame an account as this. Sir Philip says that the operation was performed in Dublin, but the text seems to shew that it was done in England.

Sir Philip's sketch is a very light and pleasant one, and the passages we have picked out will be read with amusement.

---

### Pellucid Solution of Magnesia.

This very useful and elegant preparation we have been trying for some months, as an aperient antacid, in dyspeptic complaints attended by acidity and constipation, and with very great benefit. It has the advantage over common magnesia of being completely dissolved, and therefore not liable to accumulate in the bowels. It is decidedly superior to soda or potass, on account of its aperient quality, and of its having no tendency to reduction of flesh and strength, which the two carbonates above-mentioned certainly tend to, when long continued, and taken in considerable quantities. We hope Sir James Murray, the discoverer of the process for preparing this medicine, will take the trouble to make it more generally accessible by the public in this metropolis, there being only one or two authorized agents here.

### GERMAN PHYSICIANS.

A person who signs himself J. M. and who *does* the German Schools in the Journals—one, in short, of the penny-a-liners, gives this account of German Physicians.

"It might be a curious inquiry to ascertain what are the causes of the present state of medical literature in Germany; why there is such a want of good works on medicine and surgery, along with such an abundance of excellent books on anatomy, physiology, and materia medica. Three causes suggest themselves. 1st, The theoretical and generally unpractical turn of the German mind; 2d, the want of public hospitals managed as in England; and 3d, what appears to be far the most important cause, the mode of appointment of professors in the German Universities. The aspirant to a professorship commences his career by obtaining leave to lecture under the name of '*privatim docens*.' His object is to obtain some name as soon as possible, in order to secure early promotion to a professorship. This he can most readily do by writing something; and, whether the individual has had experience in his profession or not, he writes his book. In anatomy and physiology, where every one has the means of making new observations, this is very well; but in medicine and surgery it has the most prejudicial effects. The same evil continues after the *privatim docens* has attained the rank of professor, when it is a matter of profit to him to have his system of medicine as a hand-book for his class. The general result of this is, that for practical improvements in medicine Germany has always to look to England and France, although it is not meant to be denied that from time to time works of great practical value appear."

---

### ANIMAL MAGNETISM.

Where is this celebrated *science?* What has become of "NICKEL"? Echo answers—"NIHIL"! In the whole history of human delusion there is hardly a parallel to the rapid rise, and still more rapid fall of animal magnetism in this country! It is as dead as Perkinism or the ravings of Johannah Southcote!! Is it now a wandering, disembodied spirit—viewless as the air, and silent as the grave—thus illustrating the celebrated hypothesis of one of the high priests, respecting the flights of the soul from the body? Or is it hovering about the North London Hospital and Wigmore-street, sighing over its fallen greatness, and bewailing the palmy days when it basked in the sun-shine of the LANCET? Is it still capable of pouring the balm of consolation into the wounds which its martyrs suffered, and under which they still suffer? Be this as it may, the brief career and tragic tale of animal magnetism may exercise a beneficial influence on our profession, which, alas! has proved itself—at least a portion of it—to be credulous as the ignorant African or the benighted Hindoo! Half a century hence, when the periodicals of this time are pored over by some book-worm of that day, the investigator will rub his eyes and wipe his spectacles, on turning to the pages of the Lancet and Gazette for 1838. He will scarcely believe that men, whose names, too, may go down the stream of time, with credit and honour, in other respects, should have been so infatuated as to give a moment's credence to such outrageous ravings as those of animal magnetism. Should this journal survive the ravages of time, the book-worm will find in its pages a confirmation of the fact that the MAGNETO-MANIA pervaded some portion of even the magnates of the profession in the British Metropolis! But he will learn with satisfaction, that an overwhelming majority of the profession set their faces against the imposture and scouted the credulity of their deluded brethren. The lesson has been placed on record, that such delusions cannot be participated in, or even countenanced with impunity. It is quite unnecessary to advert to the disastrous consequences which have followed in the train of this momentary mania! They will serve as a beacon to prevent the shipwreck of others.

---

### HORSE-HAIR GLOVES.

Another therapeutic agent has been lately added to the list, in the shape of "HORSE-HAIR GLOVES" for currying the human surface, when the skin is torpid—and we might add, for affording a luxurious treat to those who are afflicted with pruriginous and certain *other* cutaneous defædations attended with itching and irritation. These gloves were invented, we believe, by a gallant military officer, and possess considerable advantages over the common flesh-brush, both as to the facility of application, and the degree of counter-irritation. The cases are very numerous in which cuticular frictions are of the greatest importance, and we predict that this invention will prove very useful. In many instances it is a great desideratum to give employment to indolent patients in any kind of exercise—and that of friction is perhaps one of the best *we* can suggest, or which *they* will adopt. We observe that they may be procured at Mr. Dinneford's, Old Bond-street.

---

### CASE OF EXTENSIVE ANEURISM OF THE THORACIC AORTA. By Sir DAVID J. H. DICKSON, M.D. F.R.S.E. and L.S. Physician of the Royal Naval Hospital, Plymouth. March 5, 1839.

In the last, or 21st volume of the Transactions of the Royal Medical and Chirurgical Society, I published a case of enormous abdominal aneurism; and I now send you another extraordinary instance of extensive aneurism of the thoracic aorta. In this case and in that of Eli Hickson (aneurism of the arteria innominata) of which an abstract appeared in the 26th vol. of the Medico-Chirurgical Review, the sufferings of the patients presented a striking contrast, when compared with the state of *Keane*, above alluded to, who not only suffered so much less, but actually improved in health and flesh, during the progress of an aneurism of immense magnitude*, and they strongly illustrate the difference between a tumor confined within the walls of the chest, pressing upon vital organs, and a ventral aneurism, though of much greater size, where the yielding of the abdominal parietes and viscera, afforded such comparative facility of distention.

*Henry Ferne*, seaman, aged 35, was sent to this hospital, on the 19th of November, 1837, supposed to be in the last stage of phthisis; although attended with symptoms not usually observed in that complaint. He was stated to have repeatedly suffered from severe attacks of bronchitis—the pulse was weak and irregular, but seldom above 100—the respiration noisy and difficult—and there was some dysphagia—and a total disinclination for food (attributable, perhaps, to pressure on the eighth pair, and splanchnic nerves) and excessive emaciation.

---

* I am not aware whether there is any instance of larger, or of so large an aneurism on record as that in question. You and many of your readers are more conversant with such cases than myself: but I may observe that it appears to have much exceeded in size, that mentioned in Sir Astley Cooper's lectures, as the largest which had come under his observation; and which extended from the emulgent arteries into the cavity of the pelvis; and also (amongst others), a case of "enormous aneurism of the abdominal aorta," reported in the Lancet, alluded to by Mr. Samuel Cooper in his excellent Dictionary of Practical Surgery.

But the symptoms which most attracted attention were, the loud bronchial râles, especially the sibilous, and sonorous; pains, and a sense of stricture around and through the chest, and precordial region; severe fits of coughing, with little or no expectoration; weak, stridulous voice, and great dyspepsia, which, however, became more urgent in the afternoon. The state of the respiration might be readily accounted for, by the narrowing of the air-tubes from previous inflammation, or from the pressure of enlarged glands, or other tumor upon the windpipe, or on the nerves supplying the glottis; but I am not aware that we can depend with certainty upon any physical sign, as indicating the nature of the obstruction; as I have witnessed excessive dyspnœa, where no adequate cause of obstruction of either the circulation or respiration could be discovered *post mortem*. But it is very probable that the aneurism would have been detected, in the present instance, if there had appeared any inducement to employ auscultation on the chest and back of a patient who was in the last stage of existence.

*Sectio Cadaveris,* 22 hours P.M., presented a healthy aspect of the anterior surface of the lungs, pericardium, &c. but on raising the bronchus to ascertain the cause of the dyspnœa and stridulous voice, a large tumour was discovered behind its bifurcation; which further dissection proved to be an aneurism of the thoracic aorta, occupying the posterior mediastinum, and extending from the second to the ninth dorsal vertebræ—some of which had been rendered carious by its pressure. The dilatation commenced at the posterior part of the aorta, just below the ductus arteriosus, and, as in the case previously alluded to, by a kind of neck, which, expanding to the breadth of eight inches, descended behind the artery, œsophagus, par vagum, and other contents of the mediastinal region, as far as the diaphragm. The coverings of the aneurism, which were farther strengthened by the reflexion of the pleura anteriorly, consisted of the external and middle coats; but the internal terminated by an ill-defined line near the neck of the pouch. The walls, especially of the superior and lateral portion of the cyst, were so thin as to be ruptured by the removal of the left lung; when a large quantity of grumous blood escaped, and concentric layers of nearly colourless fibrine were separated, exposing the interior surface of the sac; which was rough, but so soft as scarcely to be distinguished from the coagulated blood. Posteriorly, where the tumour was attached to the spine, its coats had entirely disappeared by absorption, and the vertebræ (which like the heads of some of the ribs, were partially absorbed) formed the posterior boundary: but the intervertebral substance had, as usual, resisted this destructive process. The circulation was preserved through a channel in the anterior part of the tumor. The lining of the artery, for some distance below the disease, was gathered into longitudinal folds, and the normal elasticity of its tissues was diminished, although no earthy nor steatomatous deposition was detected. The heart was not diseased. The lungs were congested with frothy serum, and their texture was more friable than natural; they were not tuberculated, but the bronchial mucous membrane was evidently thickened and inflamed. The stomach and other abdominal viscera appeared to be comparatively healthy, and no further arterial disease could be detected in that cavity.

DAVID J. H. DICKSON.

P.S.—Since writing the above, I have returned from witnessing the *denouement* of a most extraordinary and puzzling case—which dissection proved to be a rupture of the duodenum; the coats of which were so extremely attenuated, that they had given way in four different places near its termination in the jejunum. The man, who had been disrated for fighting, had been seized with violent pain (which he attributed to flatulency) in the right hypochondriac region, on going to the head, at four o'clock in the morning, and which continued excruciating, notwithstanding copious depletion, and every means tried to relieve him—before and after his admission into the hospital, at 3 P.M.—and he died before midnight.

### Gleanings during Three Years spent in the Indian Seas. By W. B. Marshall, R.N.

### *Medical Establishments of New South Wales and Van Dieman's Land.*

The territory of New South Wales, at the period of my visits there in 1833-4, contained 34,505 square miles, or 22,083,200 acres of ground; and was divided into nineteen counties.

*New South Wales.*—According to a census taken on the 2nd September, 1833, there were, scattered over a space of 34,505 square miles, 60,794 inhabitants; of whom 44,643 were males, and only 16,151 females; 5,256 of the former, and 4,931 of the latter being under twelve years of age; while 22,798 male persons were free, and 21,845 bond; and 13,453 free females, with only 2,698 bond—leaving little more than one marriageable woman to three men.

The proportion of the town and rural populations, was calculated to be as one to three, or nearly so.

The Colonial Medical Department consisted of 1st. *An Inspector General of Hospitals.* 2nd. *Four Surgeons,* who were located at Sydney, Parramatta, Liverpool and Newcastle. And nine District Assistant-Surgeons, one of whom was resident at each of the following stations—Sydney, Parramatta, Liverpool, Newcastle, Windsor, Port Macquarie, Bathurst, Moreton Bay, and Norfolk Island. At each of these stations there is a Colonial Hospital; those at Sydney, Parramatta, and Liverpool, which I inspected, were more than respectable institutions, while their management was admirable and their efficiency universally acknowledged. Of that at Norfolk Island I have already published an account in the Medico-Chirurgical Review, for January 1838.

There are also hospitals attached to the two excellent Asylums for Male and Female Orphans, the former near Liverpool, the latter a little way out of Parramatta. These are attended by the district surgeons of those places, who receive extra payment for the extra duty.

There is also an hospital in the female factory of Parramatta, with a ward for midwifery cases. And an asylum for lunatics, of which also a notice will be found in the last October number of this Review.

Nosological returns from all the districts are sent in to Sydney, monthly and quarterly.

The dietary of the patients is according to a prescribed scale, varied, however, at the pleasure of the medical officer.

The allowances of the medical officers are, what the allowances of our parochial medical officers ought to be, the same as officers of corresponding ranks in the army. The apparently increased expence of this just remuneration for professional labour, is, in reality, only an enlarged economy—the health of the population being maintained by it—and the restoration to health and usefulness of a single individual being rightly considered a benefit conferred upon the community, where the population is small and the territory large—not, as in this country, looked upon as of very doubtful advantage either to the party concerned or to the public, in consequence of the received dogmas of modern political economy.

The following is an extract from a "Government Order," bearing date "Colonial Secretary's Office, Sydney, June 29, 1831;" and headed: *"Assigned Servants."* "The great expence to which the Government is subjected by the maintenance and treatment of the assigned servants of settlers, when sent into the Colonial Hospitals, having been brought under its notice, and the attention of the Government having also been called to the expence to which his Majesty's Treasury has been subjected in keeping up an extensive constabulary, a great portion of whose time has been employed in conducting the servants of settlers from Sydney to their masters in the interior; and taking others back, who, from misconduct, or, from other circumstances are returned to the Government, the following regulations have been laid down in these cases respectively, viz. :—

1. That the master shall pay at the rate of one shilling a day for the time his servant shall be in the hospital, to the extent of 30 days. Should the servant continue under treatment for any longer period, the master will not be required to make any further payment.

2. That the persons who send their servants into any of the hospitals, shall appoint an agent on the spot to take them away, as soon as they are recovered, and unless they be so taken away, they shall be considered as immediately assignable to other parties, in order to prevent the hospital from being improperly burthened with men who do not require treatment."

*Van Dieman's Land* extends from 42° to 45° south latitude, and from 145° to 148° east longitude. Its length is 210 miles, and its breadth 150, but the undulating surface of the whole island must materially increase the extent of both, taken superficially. The entire area contains 23,437 square miles. It was supposed, at the close of the year 1833, to embrace a population of 32,000 inhabitants.

For the convict portion of this population, there are, *one Surgeon*, who resides at Hobart Town, and has charge of the crowded colonial hospital in that place; *six Assistant Surgeons;* and *eleven District Assistant Surgeons;* thus making a medical corps equal in number to that of the larger and older colony of New South Wales, the population not being more than half the number of that in the latter. This numerous body being one of the many benefits flowing to the island from the philosophical and benevolent government of Colonel, now Sir George Arthur, to whose paternal anxiety for the welfare of every portion of the community, I am happy to bear testimony, from the personal knowledge I had of that truly good and great man, during my short sojourn in the island.

The 11th of his "Regulations," for prisoners of the crown, provides for the medical attendance of assigned servants in private service, on a plan, which, I should think, ensured efficient aid to every such individual throughout the colony, and ample remuneration to the medical attendant, at a cost altogether disproportioned, apparently, to the service and professional skill contracted for.

According to it, no free person can be received into any of the colonial hospitals, without an obligation being entered into by a respectable householder, making himself liable for the sum of 2s. per diem while the patient remains in the hospital, and for the expences of his funeral in case of death. The masters of assigned servants become liable for the sum of 1s. daily for one month.

The District Assistant Surgeons in the interior receive for attendance on each convict servant, five shillings per annum, payable whether sick or in health: and exclusive of medicines. Free persons in the towns, can only receive medicines gratuitously on the certificate of a clergyman or magistrate, of their inability to pay for them. And for convicts their masters must pay, as in the country, 5s. annually.

The expence to government of the entire Colonial Medical Department, including two large hospitals, was, by the above wise, humane, and just regulations, for a whole year, only £798.

I shall only remark upon those regulations, that, by them, a very necessary distinction is established between the medical skill and attendance rendered, and the medicines required to give effect to that skill and attention.

The contract of 5s. per annum on behalf of each assigned servant, sick or well, was for medical advice and assistance, Colonel Arthur rightly considering that still, they were obtained "at a very cheap rate." Medicines were to be paid for extra—except in the neighbourhood of the principal hospitals, in cases where the sick attended for relief as out-patients.

